# Comparative transcriptomics reveals suppressed expression of genes related to auxin and the cell cycle contributes to the resistance of cucumber against *Meloidogyne incognita*

**DOI:** 10.1186/s12864-018-4979-0

**Published:** 2018-08-03

**Authors:** Xing Wang, Chunyan Cheng, Kaijing Zhang, Zhen Tian, Jian Xu, Shuqiong Yang, Qunfeng Lou, Ji Li, Jin-Feng Chen

**Affiliations:** State Key Laboratory of Crop Genetics and Germplasm Enhancement, Nanjing Agricultural University, Nanjing, 210095 China

**Keywords:** *Meloidogyne incognita*, Transcriptional events, GCs, Auxin, Cell cycle

## Abstract

**Background:**

*Meloidogyne incognita* is a devastating nematode that causes significant losses in cucumber production worldwide. Although numerous studies have emphasized on the susceptible response of plants after nematode infection, the exact regulation mechanism of *M. incognita*-resistance in cucumber remains elusive. Verification of an introgression line, ‘IL10–1’, with *M. incognita*-resistance provides the opportunity to unravel the resistance mechanism of cucumber against *M. incognita.*

**Results:**

In the present study, analyses of physiological responses and transcriptional events between IL10–1 (resistant line) and CC3 (susceptible line) were conducted after *M. incognita* infection. Physiological observations showed abnormal development of giant cells and *M. incognita* in IL10–1, which were the primary differences compared with CC3. Furthermore, Gene ontology (GO) analysis revealed that genes encoding cell wall proteins were up-regulated in IL10–1 and that the highly expressed lipid transfer protein gene (Csa6G410090) might be the principal regulator of this up-regulation. Simultaneously, analyses of gene expression profiles revealed more auxin-related genes were suppressed in IL10–1 than in those of CC3, which corresponded with the lower level of indole acetic acid (IAA) in the roots of IL10–1 than in those of CC3. Additionally, poor nucleus development as a clear indication of abnormal giant cells in IL10–1 was related to inhibition of the cell cycle. Of those genes related to the cell cycle, the F-box domain Skp2-like genes were down-regulated in IL10–1, whereas more of these genes were up-regulated in CC3.

**Conclusions:**

All of these findings indicate that suppressed expression of genes related to auxin and the cell cycle and highly expressed cell wall proteins play important roles in the abnormal development of giant cells, which hinders the development of *M. incognita,* thereby causing resistance to *M. incognita* in IL10–1. Knowledge from this research will provide a useful foundation for developing effective strategies in *M. incognita-*resistance breeding.

**Electronic supplementary material:**

The online version of this article (10.1186/s12864-018-4979-0) contains supplementary material, which is available to authorized users.

## Background

Root-knot nematodes [RKN, *Meloidogyne incognita* (Kofoid and White) Chitwood] are a highly specialized type of sedentary endoparasitic plant pathogenic nematode, which are one of the very stubborn pests of vegetable crops worldwide. These nematodes penetrate at the roots tips of a host and then move upwards to the vascular cylinder, in which four to eight cells are induced into giant cells [1] as nematode feeding sites (NFS) that block transportation of nutrients in the host, resulting in reductions in yield up to 70% or more [2]. To date, strategies for controlling RKN, including nematicides and crop rotation, do not work very effectively [3]. Thus, as the most effective strategy, the breeding of plants with *M. incognita-*resistance is becoming increasingly important.

The successful invasion of *M. incognita* relies on the nutrition of the host to complete its life cycle. During the infection process, a complex defence system has evolved in plants. First, the cell wall and its reinforcement are the basal defence that hinders many types of invaders [4]. This basal defence is the first line of defence in plants and is defined as PTI (pathogen associated molecular pattern (PAMP)-triggered immunity) plus weak ETI (effector triggered immunity) which are two primary types of resistance mechanisms in the early stages of infection [5]. Previous studies demonstrate that the resistance of plants can increase by strengthening cell walls via cross linkages, lipid peroxidation, membrane damage, the activation of defence genes, and producing ROS [6–8]. Additionally, phytohormones and their signalling pathway also play an important part in plant defence against RKN at early stages of infection. Salicylic acid accumulation and the expression of pathogenesis-related (PR) genes can lead to a hypersensitive response (HR) and systemic acquired resistance (SAR) in plants against biotrophic pathogens [9, 10]. Auxins has been reported to be involved in the early development of NFS [11, 12]. Furthermore, the establishment of NFS is dependent on abnormal mitosis, which involves several cell cycle genes that are up-regulated to some extent in giant cells and syncytia based on in situ mRNA hybridization [13, 14]. However, due to few plants have *M. incognita*-resistance, studies focusing on key resistance genes are lacking, and the progress has not been sufficient towards breeding of *M. incognita-*resistant varieties. To date, a few studies have utilized next-generation sequencing technique to investigate differential host gene expression patterns during nematode-host interaction, such as rice galls and GCs, resistant soybean roots, resistant and susceptible alfalfa cultivars and common bean roots [15–20]. However, these studies are rarely reported on cucumber. Therefore, more comprehensive knowledge about the complexity of transcriptional regulation is required and the novel hub genes involved in regulating the *M. incognita* resistance must be identified in cucumber.

The discovery of cucumber line with *M. incognita* resistance is the prerequisite for solving the problems mentioned above. Fortunately, a cucumber introgression line named IL10–1 was generated by the successful interspecific hybridization between *Cucumis hystrix* and cultivated cucumber ‘Beijingjietou’ (CC3), which reported with *M. incognita* resistance [21, 22]. However, no further studies on the response of IL10–1 against *M. incognita* have been conducted to date. As an economically important specialty crop, particularly as a cultivar, the cucumber introgression line IL10–1 is the ideal material to study the resistance response of cucumber against *M. incognita* and for its application in *M. incognita* -resistance breeding.

In this study, comparative study on transcriptional events combined with the physiological responses that occurs in resistant line IL10–1 and susceptible line CC3 during *M. incognita* infection was conducted to identify the potential effector-targeted host genes and their regulation network in the IL10–1 against *M. incognita.* The information gained will be used to dissect a novel resistance mechanism in cucumber and provide a strong basis for the breeding of resistant varieties.

## Results

### Comparative galls, penetration, and development of *M. incognita* in the root system of IL10–1 and CC3

**Fig. 1:**
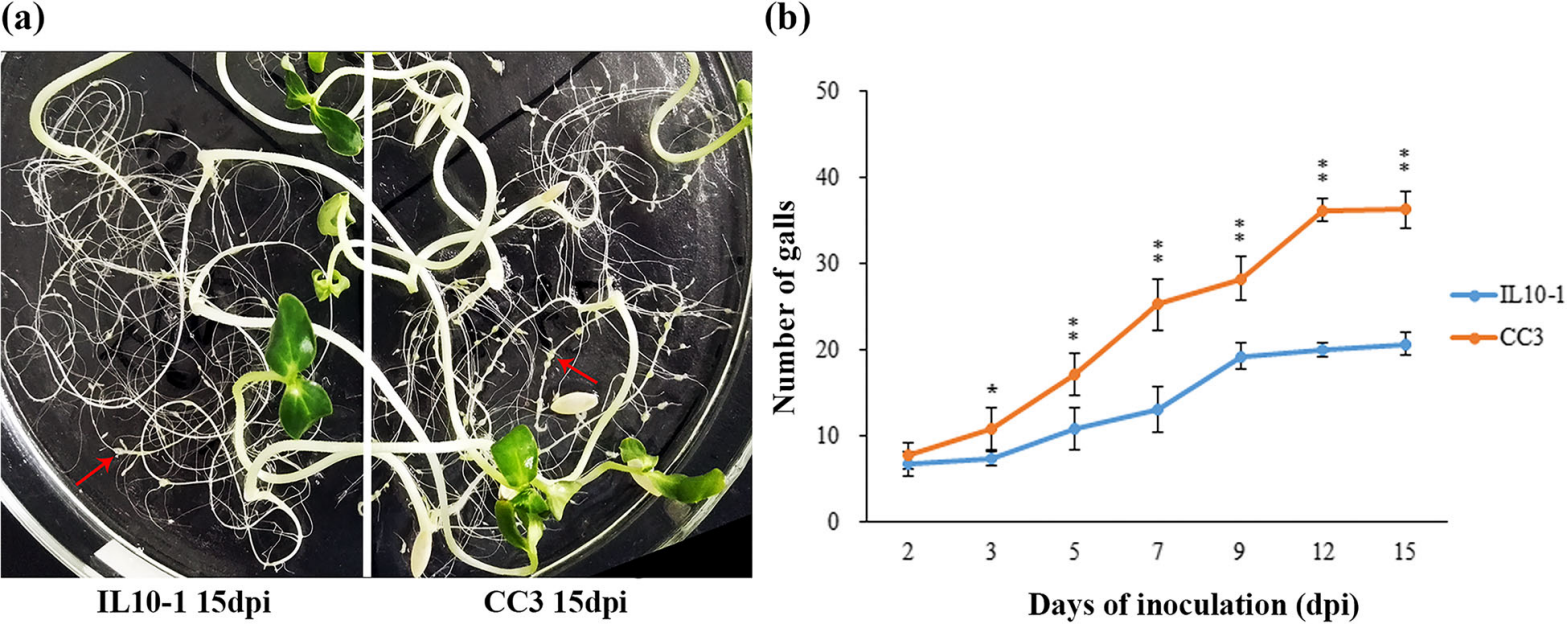
Observation of galls on the roots of IL10–1 and CC3. **a** The phenotypes of IL10–1 and CC3 at 15 dpi. The red arrows point to the galls. **b** The number of galls on roots of IL10–1 and CC3 at 2, 3, 5, 7, 9, 12, and 15 dpi. Error bars indicate standard error of the mean. Asterisks indicate significant differential expression (*, *P* < 0.05 and **, *P* < 0.01) in the comparison of IL10–1 with CC3

To confirm the resistance of IL10–1 and the susceptibility of CC3, the number of galls on roots of IL10–1 and CC3 at 2, 3, 5, 7, 9, 12, and 15 dpi was observed. The phenotypic observation showed a significant difference between IL10–1 and CC3 at 15 dpi, with more and larger galls on the roots of CC3 than on those of IL10–1 (Fig. 1a). The development process of galls was also observed. At 2 dpi the number of galls was not significantly different between IL10–1 and CC3. However, the number of galls on the roots of CC3 was significantly higher than that observed on IL10–1 roots at 3 dpi. From 5 dpi, the number of galls between IL10–1 and CC3 was highly significantly difference (Fig. 1b).

**Fig. 2:**
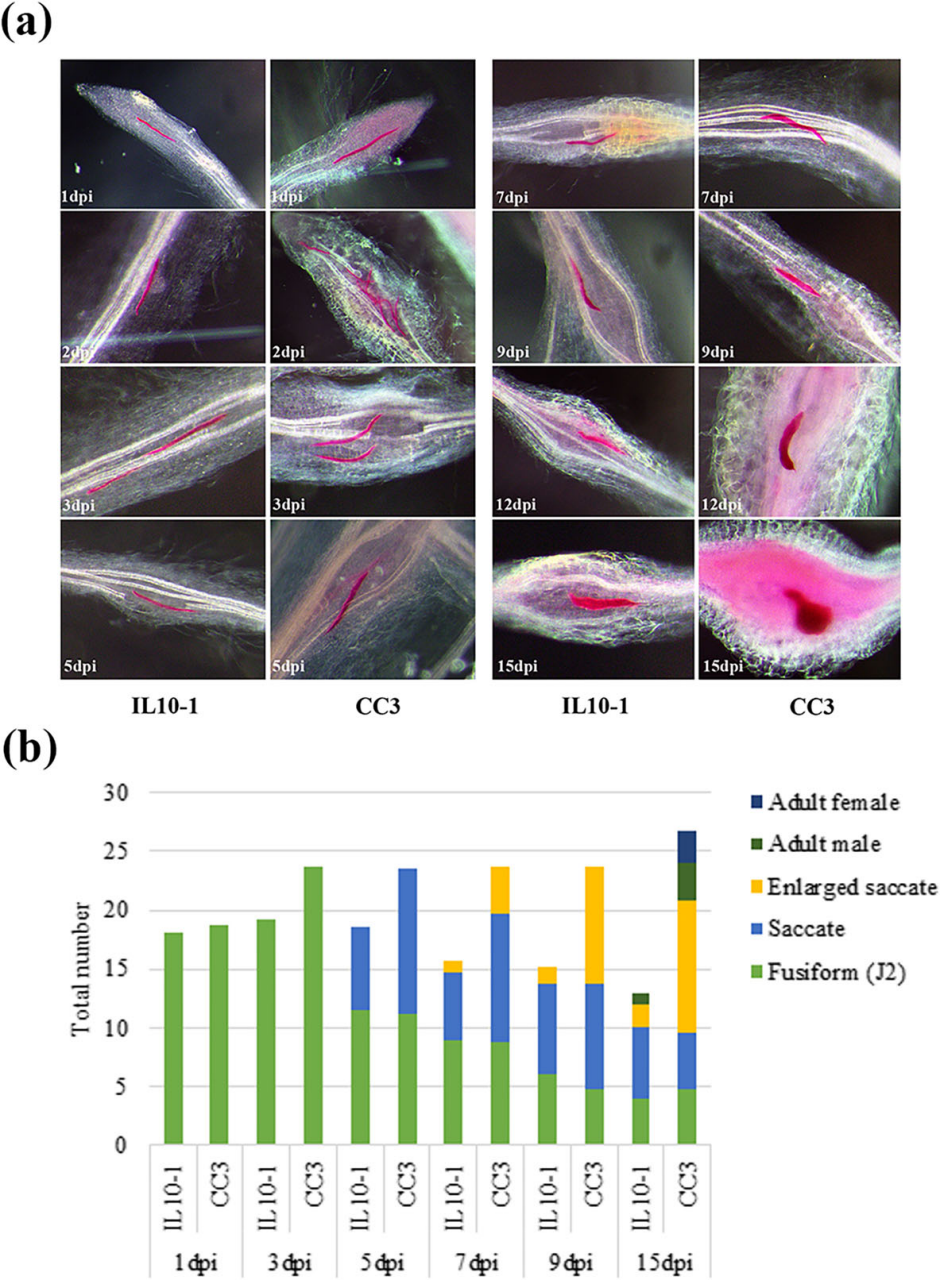
The development of *M. incognita.* **a** Penetration and development of *M. incognita* in the root systems of IL10–1 and CC3. **b** The number of nematodes at different developmental stages after J2 inoculation

To further reveal the differences between IL10–1 and CC3, penetration and development of *M. incognita* in IL10–1 versus CC3 were observed by staining with acid fuchsine. The infective J2 s first invaded from the root tip and then moved up along the vascular cylinder. Ultimately, some suitable cells were selected as the feeding site (Fig. 2a). Analysis of the course of *M. incognita* infection in the root systems of IL10–1 and CC3 showed that the initial penetration of infective second-stage juveniles (J2) was similar in both materials (Fig. 2b). At 1 dpi, the number of J2 s in IL10–1 was similar to that in CC3. However, compared with IL10–1, the number of J2 s in CC3 increased substantially at 3 dpi. From 3dpi, a downward trend in the total number of nematodes was only found in IL10–1. Notably, the development of fusiform J2 s was hampered in IL10–1 compared with that in CC3. As a result, the adult female and male nematodes were barely apparent in IL10–1 at 15 dpi.

### Statistics of transcriptional sequencing

To obtain insight into the changes in gene expression levels during the early infection stages, RNA was isolated from root tips of IL10–1 and CC3 at 0, 1, 2 and 3 day post inoculation (dpi), with three biological replicates. The RNA was then subjected to whole transcriptome sequencing via RNA-seq. Approximately 46.18 million 150 bp paired-end clean reads per sample were obtained after cleaning and checking the reads quality. Approximately 98% of clean reads were aligned uniquely to the cucumber genome using the software TopHat (Additional file 1: Table S1). Then the expression level of each gene was calculated using RPKM. The correlation clustering among the three biological replicates of each sample was conducted based on the expression level of all genes. All biological replicates showed correlation coefficients above 0.9 indicating good reproducibility between biological replicates (Additional file 2: Figure S1).

### Differences in gene expression patterns in resistant IL10–1 and susceptible CC3

**Fig. 3:**
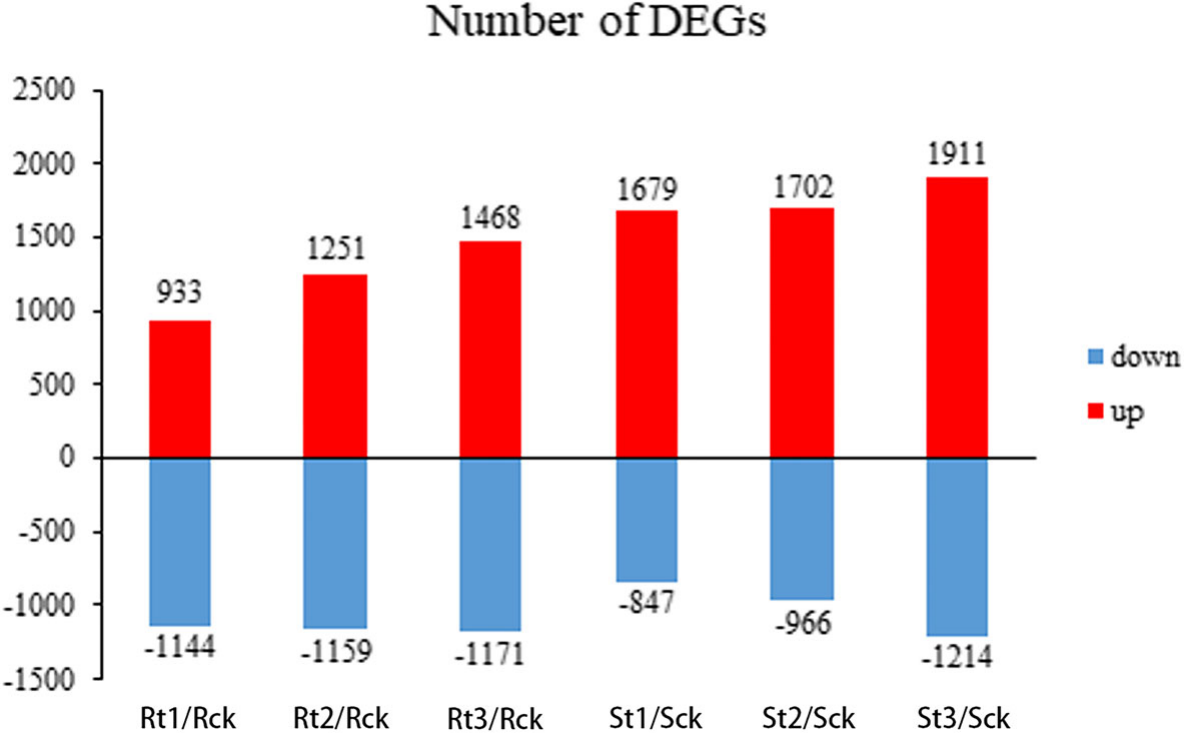
The numbers of differentially expressed genes at different stages of infection with *M. incognita* in IL10–1 and CC3 compared with those in control. Up indicates up-regulated genes, Down indicates down-regulated genes

Statistically significant DEGs, with a false discovery rate (FDR) less than 0.05 and a fold change greater than 2, were identified by the DESeq R package among inoculated samples compared with respective non-inoculated controls, across the three investigated time points. Thus, the different expression patterns of the two genotypes were revealed (Fig. 3).

To specifically analyse the differences in expression patterns, the *k*-means clustering of the gene expression patterns of the two genotypes was performed. The gene expression modes were classified into 17 subclusters, which showed more specific and diverse expression patterns (Additional file 3: Figure S2). Most of the subclusters shared similar expression patterns in both IL10–1 and CC3, except for the subclusters 6, 9, 13 and 16. Most genes in subcluster 6 were up-regulated in CC3 at 1 and 2 dpi, which was in contrast to the gene expression patterns observed in IL10–1. In subcluster 9, the pattern showed an up-regulated mode in IL10–1, whereas CC3 showed a smooth line, indicating these genes positively responded to RKNs, particularly in IL10–1. In subcluster 13, compared with the smooth line in IL10–1, CC3 showed a down-regulated pattern suggesting more genes were suppressed in response to RKNs infection. Subcluster 16 showed the exactly opposite pattern to that in subcluster 13, with a down-regulated pattern in IL10–1, but a steady trend in CC3.

**Fig. 4:**
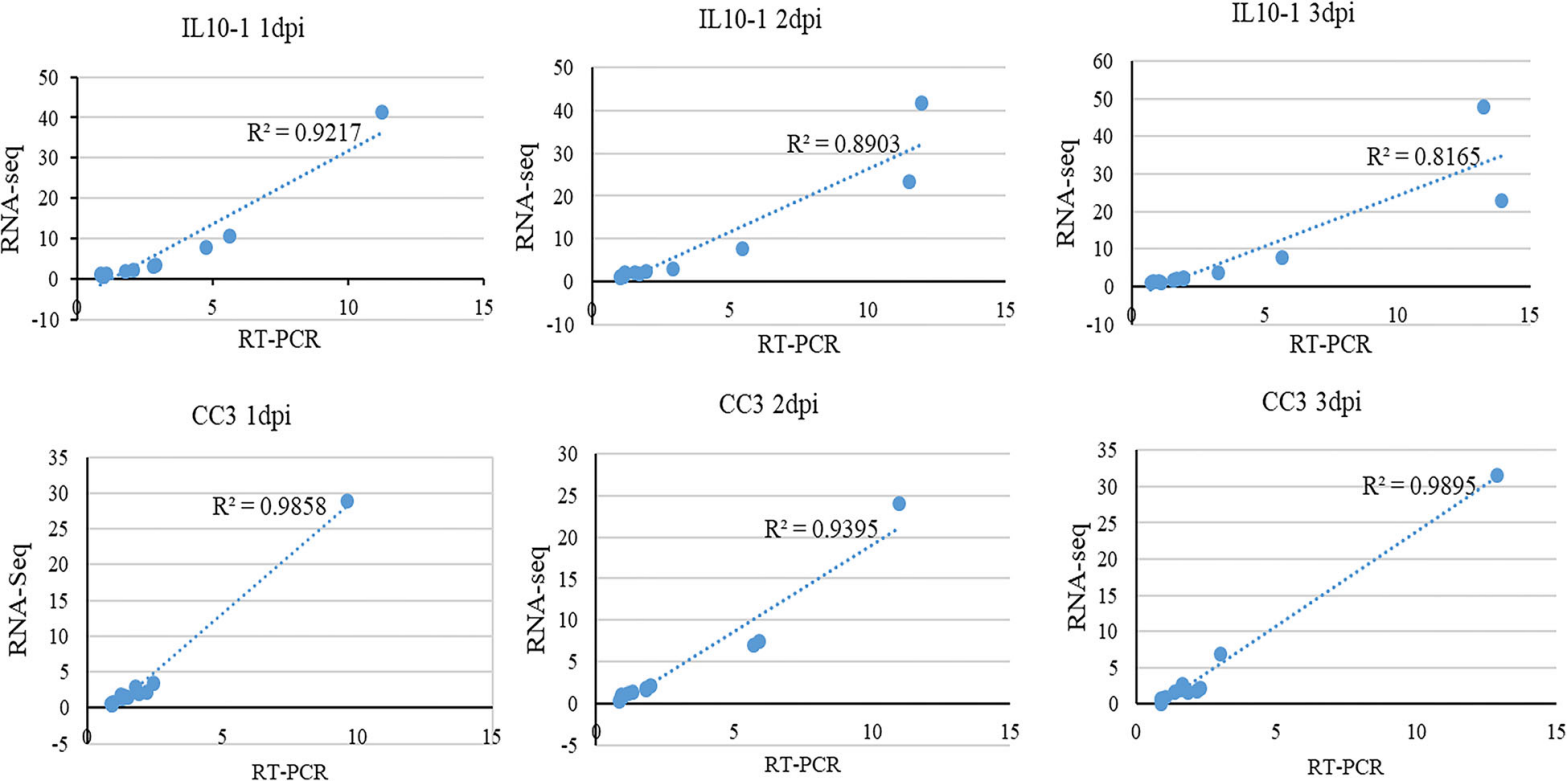
Verification of the expression profiles of 12 genes obtained in RNA-seq analysis by qPCR. The RNA-seq log2 value of the expression ratio (inoculated/mock-inoculated) (y-axis) was plotted against the value from the RT-PCR (x-axis). Dpi indicates days post inoculation

To verify the validity of these results, twelve genes with altered expression levels were chosen for quantitative real-time RT-PCR (q-PCR). The same expression patterns and the excellent Pearson’s correlations between q-PCR and RNA-seq data indicated high reliability of the RNA-seq results (Fig. 4).

### Functional categorization of DEGs in subclusters

**Fig. 5:**
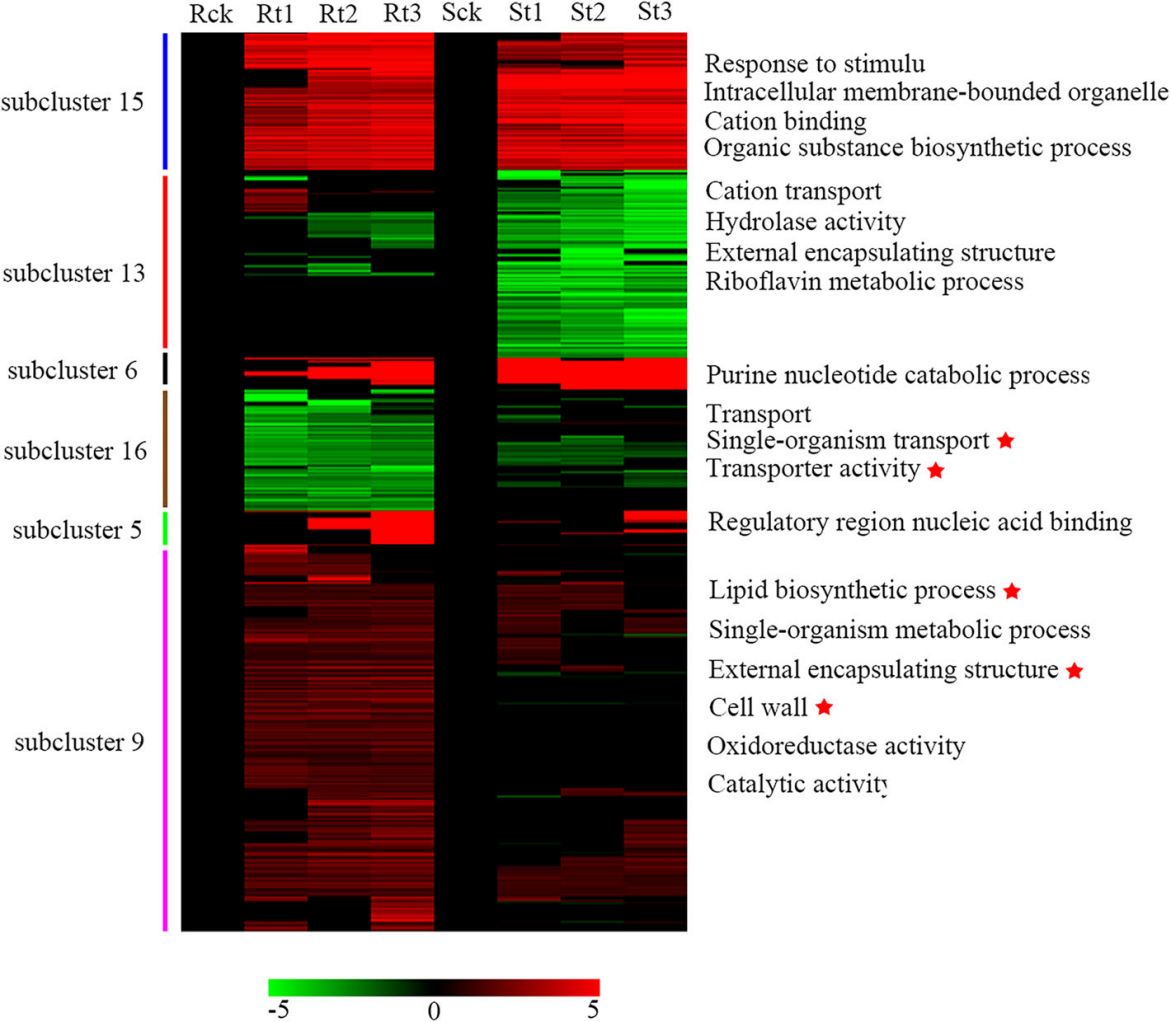
Gene expression pattern and functional categorization over the time course of infection with *M. incognita* in IL10–1 and CC3. Heat maps showing the expression patterns of genes in 6 selected K-means clusters in IL10–1 and CC3. The value of each gene shown is the log2 FC compared with that of the control. Red indicates up expression; whileas green indicates down expression. Overrepresented (FDR < 0.05) functional categories are indicated on the right

Based on the putative functions assigned by the Cucumber Genome Annotation Project of DEGs in the different subclusters in response to *M. incognita*, GO enrichment analysis was performed using the topGO. For each subcluster, all GO terms were attributed to the following three categories: biological process, cellular component and molecular function (Additional file 4: Table S2). In subclusters 1, 3, 5, 15 and 17, which showed an upward trend, the enriched GO terms with the most DEGs were belonged to response to stimulus and metabolic process classified in the biological process category and to the cell component subcategories: plastid thylakoid and organelle subcompartment. Those GO terms might suggest the general response or the basic roles in responding to *M. incognita*. Considering the different phenotypes under the infection of *M. incognita* and identifying the key features of resistance, divergent patterns of expression between resistant IL10–1 and susceptible CC3 were the focus. For example, subcluster 13 showed no significant changes in IL10–1 but presented a downward trend in CC3 (Fig. 5), and in this subcluster, cation transport, hydrolase activity and external encapsulating structure were enriched. Perhaps, the inhibition of these genes resulted in the increased susceptibility of CC3 to *M. incognita*. In subcluster 16, single-organism transport and transporter activity were suppressed in IL10–1, which might cut off the inflow of nutrition to *M. incognita*. More noteworthy, subcluster 9 presented an upward trend in IL10–1; whereas the subcluster was stabilized in CC3. The genes related to external encapsulating structure, cell wall, lipid biosynthetic process and catalytic activity were enriched, indicating that these genes might positively regulate the IL10–1 resistance to *M. incognita*. These results contributed to the identification of functional genes underpinning IL10–1 resistance and to the understanding of the different mechanisms that IL10–1 and CC3 responded to *M. incognita.*

### Co-expression network analysis and identification of hub genes with *M. incognita* resistance

To reveal the interaction between genes directly and identify the hub genes in the centre of the regulatory network in the defence against *M. incognita*, an alternative analysis tool, WGCNA, was adopted. The samples with significant differences in gene expression from 0, 1 and 3 dpi of both IL10–1 and CC3 were selected for co-expression networks construction. A total of 21 modules with different colours were obtained based on the correlations between genes in their common expression trends. Of the modules, the MEgrey modules with correlation coefficients below 0.85 were eliminated from further analysis. The cluster dendrogram (Additional file 5: Figure S3a) showed that each tree branch constituted a module and each leaf in the branch was one gene. By analyzing the correlation between samples and each module eigengene that as the first principal component of each module represented the module’s gene expression profile, the module with the strongest relationship with the sample was used in further study (Additional file 5: Figure S3b).

**Fig. 6:**
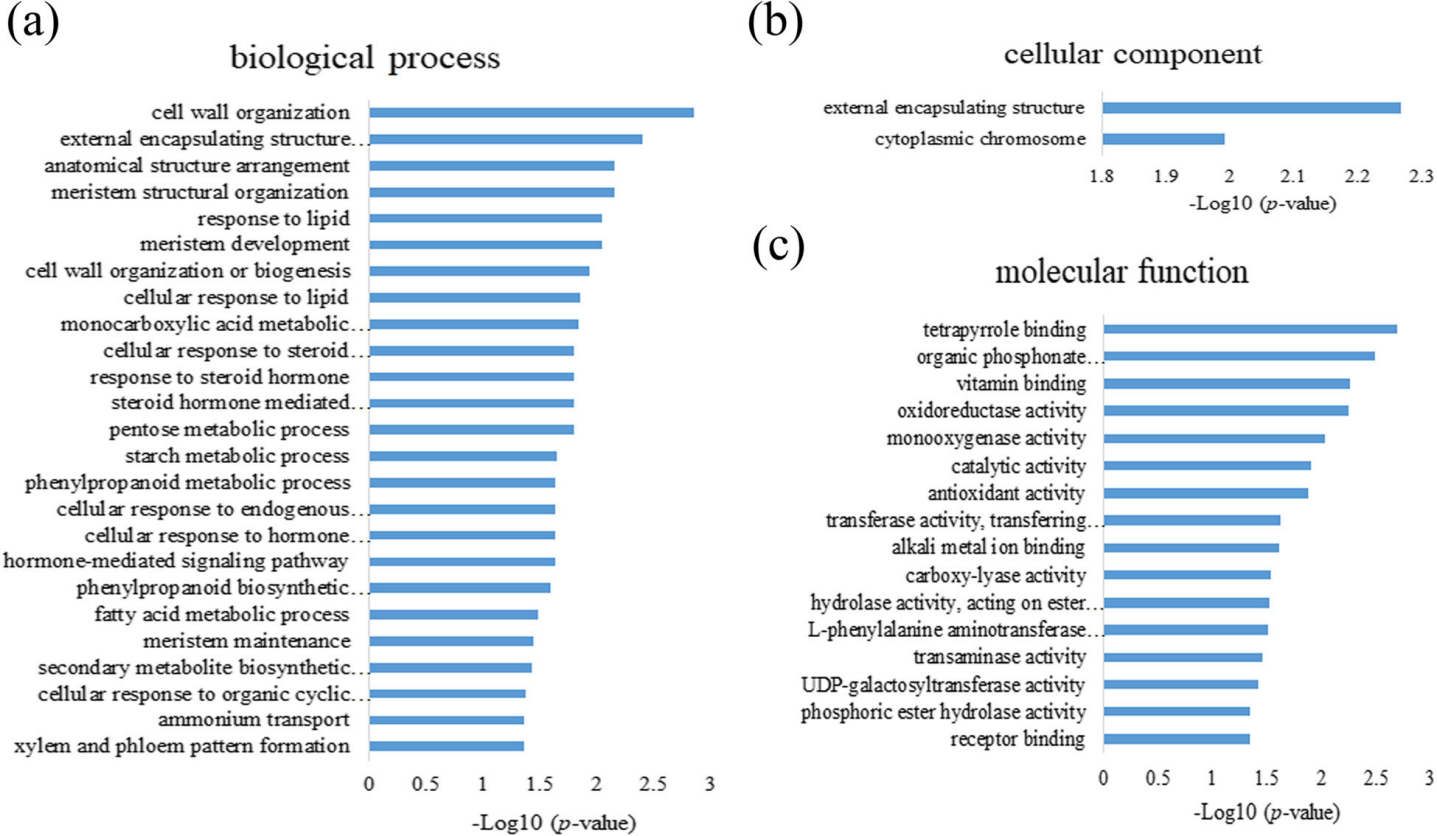
GO term enrichment of the specially induced genes in IL10–1 according to the subcluster 9 of K-means. **a** Genes enriched in biological process. **b** Genes enriched in cellular component. **c** Genes enriched in molecular function

**Fig. 7:**
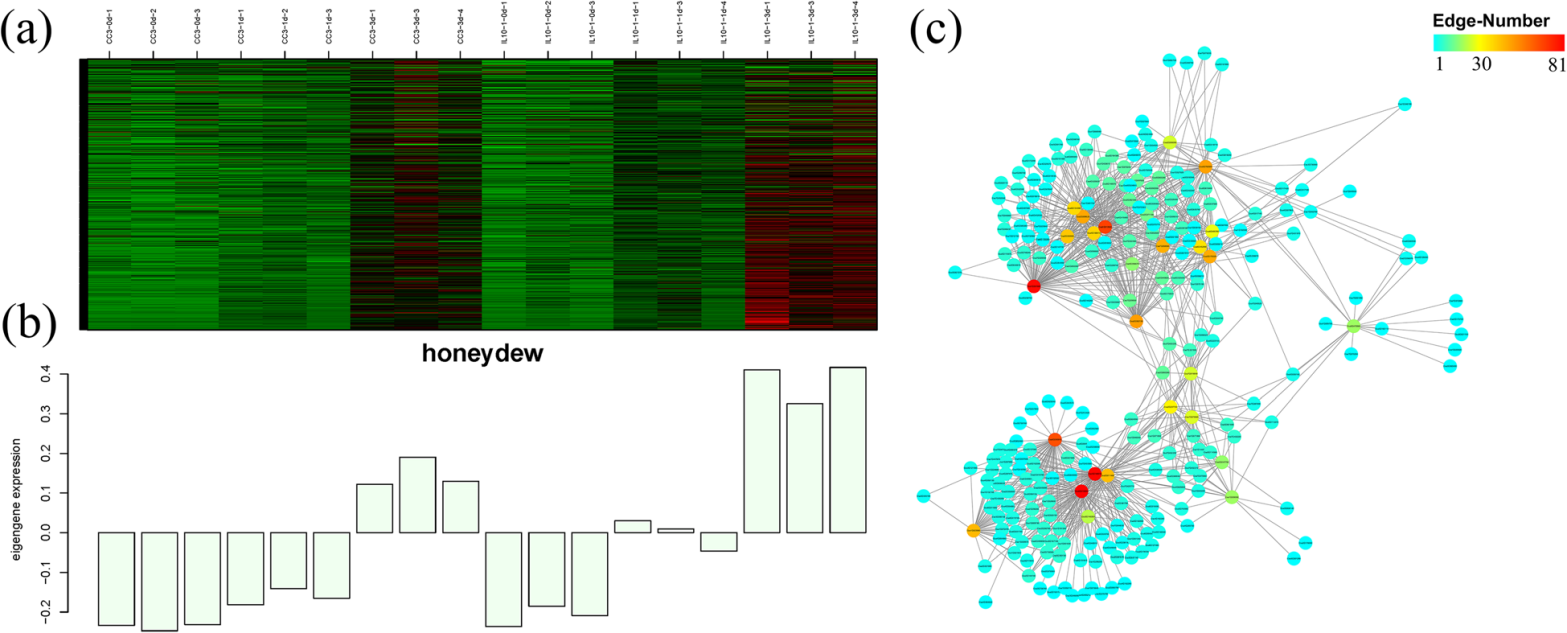
The expression and network of genes in the MEhoneydew module. **a** Heat map showing the relative RPKM of each gene from the MEhoneydew module. The red and green represent higher and lower expression levels, respectively. **b** Eigengene expression profile for the MEhoneydew module in samples from different inoculation stages. The y axis indicates the value of the module eigengene; the x axis indicates the samples. **c** The co-expression network of the MEhoneydew module with the edge weight higher than 0.15 visualized by Cytoscape. The red and blue represent higher and lower edge numbers, respectively

Of particular interest was the sample of IL10–1 at 3 dpi which had a significant difference in phenotype compared with that of CC3 (Figs. 1,and 2). Notably, the MEhoneydew module with a relatively high relationship with IL10–1 was selected. The heat map showing the relative RPKM of each gene from the MEhoneydew module and eigengene expression (Fig. 7a, b) revealed that the strongest expression of genes of the sample from IL10–1 at 3 dpi. Consistent with analysis results using K-means clustering, GO enrichment analysis (Fig. 6) of those genes showed that external encapsulating structure, hormone-mediated signalling pathway, cell wall and catalytic activity were primarily enriched, indicating that the molecular function of these genes played a vital role in responding to *M. incognita*.

As one of the advantages, genes co-expression network constructed by WGCNA, with each node representing a gene and the connecting lines (edges) between genes representing co-expression correlations, can be employed to identify the hub genes that present the most connections in the network. In total, 259 genes with an edge weight higher than 0.16 were visualized in the MEhoneydew module network. The results showed (Fig. 7c; Additional file 6: Table S3) that the gene Csa6G410090 annotated to plant lipid transfer protein (LTP) with the highest edge number (85 edges) was the hub gene. As a member of plant pathogenesis-related protein (PRs), it plays an active role in a variety of plant resistance reactions; thus, the result highlighted Csa6G410090 as a possible key regulator in responding to *M. incognita*. Other highly connected hub genes are listed in detail in Table 1. The identification of these hub genes could be a powerful means for the location of resistance genes in IL10–1 used in defence against *M. incognita*.

**Table 1:** The eight hub genes in the MEhoneydew module. RPKM indicates the log2 value of the expression ratio (inoculated/mock-inoculated). R: IL10–1, S: CC3

Gene ID	RPKM				Edges	IPR-Description
	Rt1/Rck	Rt3/Rck	St1/Sck	St3/Sck		
Csa6G410090	20.49	58.54	3.72	11.57	85	IPR003612 Plant lipid transfer protein
Csa5G148510	7.92	25.57	2.23	7.89	84	IPR000073 Alpha/beta hydrolase fold-1
Csa1G597800	4.67	7.94	1.82	5.48	83	IPR001296 Glycosyl transferase group 1
Csa6G013920	51.77	100.73	6.38	23.79	71	IPR007667 Hypoxia induced protein domain
Csa6G095910	2.82	5.33	3.65	6.46	68	IPR017930 HTH transcriptional regulator Myb-type DNA-binding
Csa2G302130	1.79	2.63	1.10	1.92	51	IPR006501 Pectinesterase inhibitor
Csa3G760500	4.27	8.90	1.17	4.37	50	IPR013770 Plant lipid transfer protein
Csa2G009620	17.49	27.53	3.28	10.21	49	IPR005123 Oxoglutarate/iron-dependent oxygenase

### Response of genes related to auxin and the cell cycle in defence against *M. incognita*

**Fig. 8:**
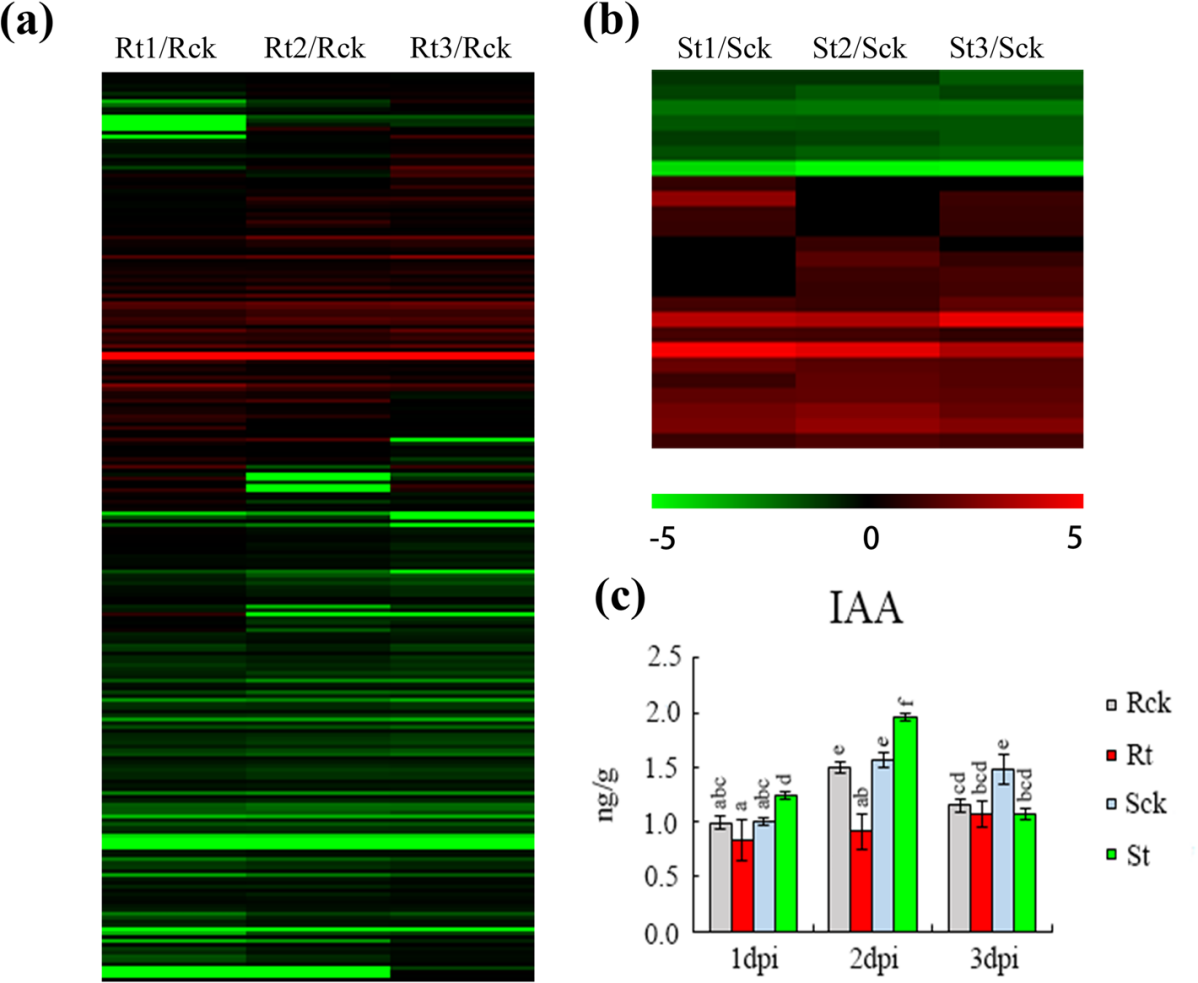
Expression of auxin-related genes and quantification of IAA at different processing times in both IL10–1 and CC3. **a, b** Heat map showing the expression of auxin-related genes. The value of each gene shown is the log2 FC compared with that of the control. Red indicates up expression, whileas green indicates down expression. **c** Quantification of IAA in the inoculated or mock-inoculated plants of both IL10–1 and CC3

Phytohormones are well documented with wide involvement in the host plant response to RKN infection. In this study, auxin-related DEGs showed significant differences between IL10–1 and CC3. The number of DEGs in IL10–1 was clearly greater than that in CC3, many auxin-related genes were significantly down-regulated in IL10–1 (Fig. 8a, 6b). These genes included homologous genes of indole-3-acetic acid-amido (IAA) synthetase GH3 and auxin response factor (ARF), which are the major genes involved in auxin biosynthesis and signalling pathways. These results indicated that auxin biosynthesis and signalling pathways were inhibited more strongly in IL10–1than in CC3.

To confirm the above results, the contents of IAA in inoculated or mock-inoculated plants of both IL10–1 and CC3 were measured (Fig. 8c). At 1 dpi, for the mock-inoculated plants, the content of IAA was not significantly different between IL10–1 and CC3; however, after inoculation, a significant difference occurred with IAA lower in IL10–1 and higher in CC3. This phenomenon was more significant at 2 dpi, and of all sample times, the content of IAA reached the highest value in CC3 at this point. At 3 dpi, the level of IAA was similar in IL10–1 and CC3. These results were consistent with the gene expression data that suggested the inhibition of auxin expression plays important roles in plant defence against *M. incognita*.

**Fig. 9:**
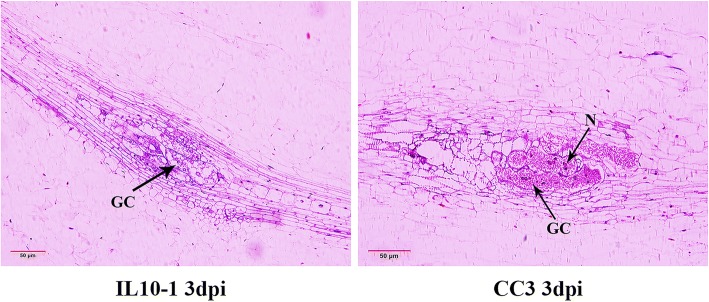
Longitudinal sections of nematode feeding sites in IL10–1 and CC3 at 3 dpi. GC indicates giant cells, N indicates nucleus. The red lines as a ruler represent 50 μm

Auxin has been reported to be involved in the formation of giant cells [23]. Indeed, in this study, IL10–1 showed fewer and smaller galls than those of CC3 (Fig. 1). To distinguish the histological differences of GCs between IL10–1 and CC3, paraffin sections of feeding sites in IL10–1 and CC3 at 3 dpi were conducted. In contrast to the more nucleus and dense cytoplasm in the GCs of CC3, the GCs of IL10–1 showed no clearly visible nucleus and a thinned cytoplasm concentration (Fig. 9), which might be caused by the inhibition of auxin expression.

**Fig. 10:**
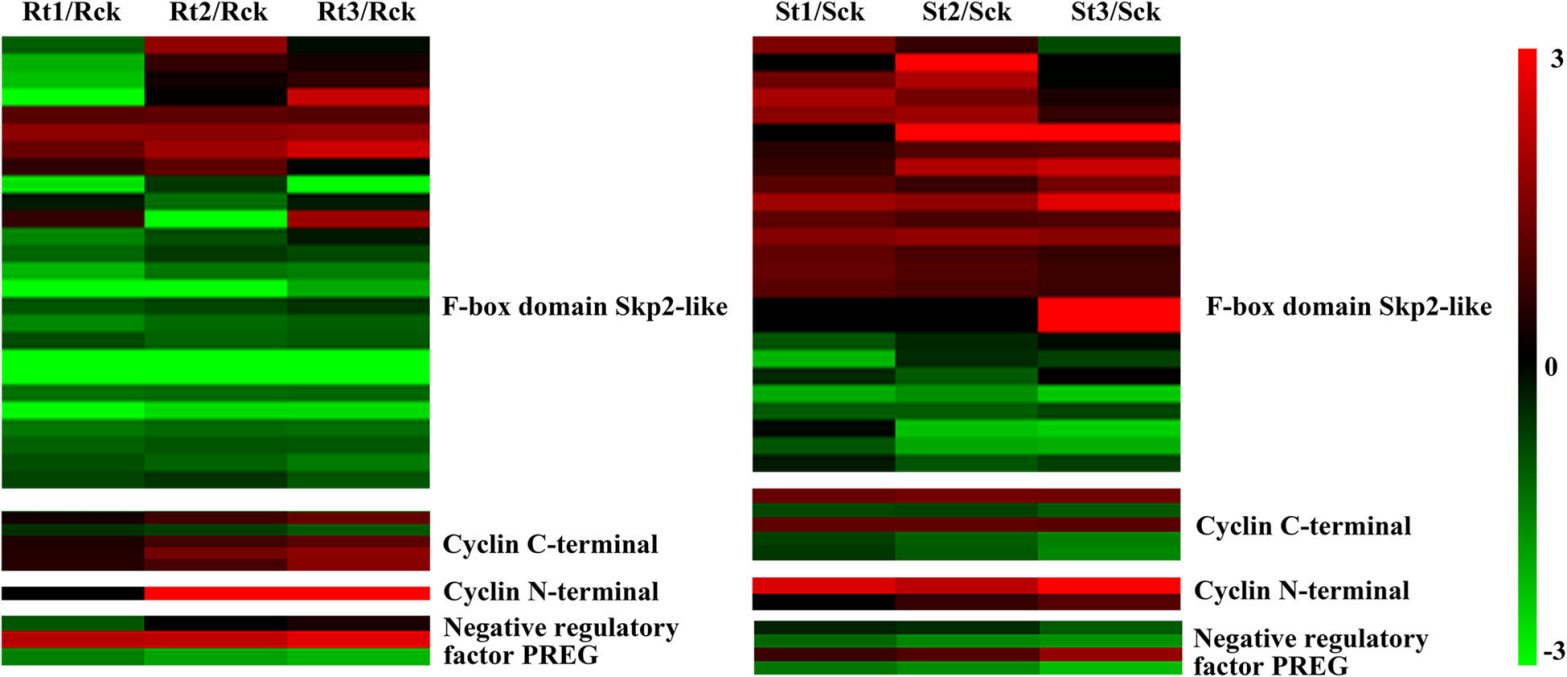
The expression profiles of all cyclin genes of the DEGs in IL10–1 and CC3. The heat map shows the value of the log2 FC of each cyclin gene compared with that of the control. Red indicates up expression, whileas green indicates down expression. Annotation of each gene is described on the right

Multicellular nucleus is the primary feature of GCs induced by RKNs. The basis for nucleus differentiation involving some cell cycle genes is endoreduplication. Gheysen [24] showed the cell cycle is involved in regulating the formation of feeding sites. Thus, all cyclin genes of the DEGs in both IL10–1 and CC3 were selected for further analysis. Comparative analysis of the expression of cyclin genes in IL10–1 and CC3 showed that F-box domain Skp2-like genes were suppressed in IL10–1 during the infection of *M. incognita*, particularly at 1 dpi compared with those in CC3 (Fig. 10). These results indicated that the phenomenon of fewer nucleus that appeared in IL10–1 might be caused by the inhibition of the cyclin genes, particularly that of the F-box domain Skp2-like genes.

## Discussion

### The abnormal development of GCs leading to suppressed or delayed development of *M. incognita* is the primary resistance characteristic of IL10–1

As the first line of plant defence, hampered penetration in resistant plants is a relatively common phenomenon in plant-nematode interactions, such as occurs peanut and coffee against nematodes [14, 25]. However, our results showed that it did not preclude nematode penetrating into the root of IL10–1, which might be due to the absence of physical and/or chemical barriers similar to resistant alfalfa and Lotus japonicus [18, 26]. The substantial difference that occurred between two genotypes was in the development of nematodes that had penetrated in roots. In *Cucumis metuliferus,* reported with *M. incognita* resistance, the infecting nematodes were surrounded by t hypersensitive necrosis cells, which seriously hindered nematode development [27]. In this study, in contrary to that observed in CC3, the total number of nematodes began to decline in IL10–1 from 3 dpi (Fig. 2b). Over time, the nematodes in roots of IL10–1 were small and poorly developed compared with the relatively perfect development status of nematodes in CC3 (Fig. 2b). This phenomenon also occurs in resistant *C. sativus var hardwickii,* which hinders nematode development compared with susceptible cucumber [28]. Additionally, no females were in roots of IL10–1 compared with a few in CC3 (Fig. 2a, b). This result is similar to that found in cotton isoline 81–249 in which inhibit initial J2 is not inhibited but further development into reproductive females is suppressed [29]. This phenomenon was possibly due to the lacking of nutrition. It had been reported that limitations in nutrient supply not only affect the development of nematodes but also determine sexual differentiation. Juveniles of *Meloidogyne floridensis* develop into females under normal nutrient conditions but into males when lacking nutrients [30].

As known, NFS are the source of nutrients for the developing of sedentary nematodes within the root tissue. The elongated giant cells observed in *C. sativus var hardwickii* are identified as the primary resistant characteristic [28]. Therefore, the normal development of GCs is the basis for nematode development. The normal GCs with more nucleus and dense cytoplasm observed in CC3 provided the nutrients required for *M. incognita* growth and development (Fig. 9) [1, 15]. However, the development of GCs was inhibited in IL10–1 with a thinned cytoplasm concentration and poorly developed nucleus, which is the first resistance characteristic observed in cultivated cucumbers. Similar features appear in cowpea cultivar C-152, which is less resistant or moderately resistant to *M. incognita* [31]. Additionally, GO enrichment analysis of the DEGs in subcluster 16 showed single-organism transport and transporter activity were enriched in IL10–1 compared with those in CC3 (Fig. 5). Many genes involved in transport and allocation in nematode-induced syncytia have been analyzed by transcriptome [32, 33]. Based on T-DNA insertion mutants and RNAi-based gene silencing, genes related to sugar transporters are identified with a significant role for nematode development [34, 35]. However, few previous studies focus on the transport in *M. incognita*-induced GCs. In this study, genes of ATPase P-type H+ transporting proton pump (Csa7G379080) and Amino acid transporter transmembrane (Csa4G361780) were significantly inhibited, which might cut off the nutrient supply to *M. incognita.*

Additionally, cell walls and their reinforcements can withstand any types of invader [4]. Callose deposition, lignification and ROS production, which strengthen the cell wall structure, were activated with BABA (β-aminobutyric acid) treatment, resulting in delayed development of nematodes [36]. GO enrichment analysis of the DEGs that were up-regulated in IL10–1 in subcluster 9 showed that genes related to external encapsulating structure, cell wall, lipid biosynthetic process and catalytic activity were enriched in IL10–1(Fig. 5). In this study, 41 DEGs related to cell walls and external encapsulating structure were detected in subcluster 9 during infection, including Csa2G302130 annotated to pectinesterase inhibitor, which may contribute to decrease the deacetylation of pectin, a major compound of primary cell walls [37]. Therefore, we hypothesized that the abnormal development of GCs, the cell walls reinforcement and the suppressed transport that lead to a lack of nutrients inhibited the development of *M. incognita* in IL10–1.

### The LTP genes with the highest expression were identified as the hub genes by WGCNA

Through the construction of a co-expression network, 8 genes with the highest edges were considered the core genes of the network and assumed to have great roles against *M. incognita* (Table 1). One gene, Csa2G302130, was also identified by K-means analysis, indicating the reliability of those results. Among the 8 genes, 2 genes that were related to plant lipid transfer proteins (LTPs) were significantly induced; one of which had the most edges and was considered as the hub gene. Oda Y. et al. found that LTPs of wheat can inhibit α-amylase and therefore contribute to resistance against biotic stress [38]. Although an increasing number of studies had demonstrate that LTPs are associated with plant resistance and defence [39, 40], the exact mechanism remains unclear. According to Jiang et al., LTPs can promote the synthesis of cell surface wax and increase the thickness of the stratum corneum, forming a mechanical barrier [41], whereas Maldonado et al. speculated that LTPs are involved in the signal transmission of plant system acquired resistance [42]. Studies on the relationships between LTPs and plant nematodes are rarely reported, and therefore, the detailed response mechanism requires further study. Additionally, one gene that annotated to Myb-type DNA-binding transcriptional factor was also identified that regulates the downstream gene expression, including that of the LTPs. Previous studies show that LTP3 is positively regulated by the transcription factor MYB96 to mediate freezing and drought stress in Arabidopsis [43]. Whether there is a link between Myb type DNA-binding transcriptional factor and LTP during the plant-pathogen interaction process remains to be verified.

### Suppressed expression of genes related to auxin and the cell cycle lead to the abnormal development of GCs in IL10–1

Previous studies demonstrate that both *M. incognita* and the potato cyst nematode (PCN: *Globodera rostochiensis*) are impaired in their development in auxin insensitive mutants because of the arrest in early feeding cell formation [44–46]. GH3 acts as an auxin-responsive promoter that is rapidly and transiently activated during root gall initiation by *Meloidogyne* [23]. However, our results showed that most homologous genes of GH3 were suppressed in IL10–1. Consistent with the auxin-related genes, the IAA concentration remained at a lower level during the infection in resistant line IL10–1 than that in the control, whereas the IAA concentration was significantly higher in susceptible line CC3 than that in the control, particularly at 2 dpi.. Thus, we conjectured that the lack of auxin contributed greatly to the abnormal development of GCs in IL10–1 that are observed in Fig. 9, thereby cutting off the feeding source of *M. incognita.*

The reduced nucleus observed in IL10–1 as an intuitive feature was an important factor for the abnormal development of GCs. Expression and activation of a significant number of core cell cycle genes are involved in regulating the cell cycle in NFS [47, 48]. Analysis of the expression of cyclin genes showed that most F-box domain Skp2-like genes were suppressed in IL10–1, whereas these genes were up-regulated in CC3 (Fig. 10). The F-box protein Skp2 is important for S phase entry and binds to Skp1 and the cyclin A-Cdk2 complex [49]. It had been showed a SKP1-like protein played a role in the GCs formation [50]. Nuclear DNA is replicated during the S phase, and the successful establishment of GCs requires repeated passes through the S-phase, which is the premise for the nucleus division in the M phase. It is likely that the abnormal development of GCs was caused by the inhibition of F-box domain Skp2-like genes.

Furthermore, it has been reported that auxin and cell cycle had a certain interaction. The auxin-regulated cell cycle genes *AtCDC2a* and *AtCYCB1;1* are among the earliest genes activated during feeding sites initiation [48, 51]. Although no reports are available about the interaction of auxin and F-box protein Skp2 genes in regulating GCs morphology, control of F-box protein Skp2 gene stability by auxin-dependent degradation in regulating cell division has been confirmed [52]. However, the exact mechanism by which auxin and F-box protein Skp2 genes cooperate to regulate GCs morphology must be further revealed.

## Conclusions

Overall, studies on the cucumber line ‘IL10–1’ identified with *M. incognita* resistance, indicated that suppressing or delaying the development of *M. incognita* was the primary resistance characteristics. And the identification of hub genes by WGCNA showed that LTPs might contribute to the resistance of IL10–1. Comparison of transcriptomes revealed that auxin and cell cycle genes were inhibited, which caused the abnormal development of GCs, and finally resulted in the blocking of *M. incognita* development in IL10–1. These results provided novel insights into the understanding of major resistance features and the key molecular mechanisms in plant against *M. incognita*.

## Methods

### Plant materials, culturing of nematodes and inoculation

Two individuals that display marked differences in against *M. incognita*, the resistant line IL10–1 and the susceptible line ‘beijingjietou’(CC3) [21], provided by the state key lab of Cucurbit Genetics and Germplasm Enhancement of Nanjing Agricultural College, were used in the experiments. Before germination, seeds of both were surface sterilized with 70% ethanol for 15 s and with 1% sodium hypochlorite solution for 10 min, followed by rinsing with distilled water three times. Sterilized seeds were placed on wet filter paper inside Petri dishes and incubated in a growth chamber at 28 °C. Seedlings of 3–4 days old were used for the following experiments.

Inoculum of *M. incognita* race 1 was prepared by separating nematode eggs from infected roots of tomato plants ‘Hezuo 903’ planted in greenhouse of Nanjing Agricultural College. It is the dominant species in most parts of China and kindly provided by the institute of plant protection, Nanjing Agricultural University [53]. Referring to the method of Hussey R. S., fresh and motile second-stage juveniles (J2) were collected as inoculum [54].

Pluronic F-127 (PF-127) (Sigma-Aldrich, USA) gel was prepared as a medium for RKN infectivity [55, 56]. Eighty millilitres of 23% PF-127 was poured into each Petri dish (150 mm, wuyi) containing 12 uniformly distributed seedlings of the identical variety at 15 °C. Before transferring to room temperature, approximately 50 J2 s of *M. incognita* race 1 were inoculated at the root tip of each seedling using a pipette tip. Then, each Petri dish wrapped with clinging plastic wrap and with several holes was placed in a humidity tray and incubated at 28 °C with a 16:8 h light: dark photoperiod in a growth chamber. One-day post inoculation (1 dpi), seedlings were rinsed with distilled water and transplanted in wet filter paper with Hoagland’s nutrition under the same conditions as above.

### Nematode development assays

For phenotype identification, the number of root galls on IL10–1 and CC3 harvested at 2, 3, 5, 7, 9, 12, and 15 dpi was counted. Each sample had repeated at least 7 replicates. Using the same inoculation method, the roots of the two cultivars were stained with acid fuchsin at 1, 2, 3, 5, 7, 9, 12 and 15 dpi respectively [57]. The average number of nematodes in different physiological states at 1, 3, 5, 7, 9 and 15 dpi was counted and the development of the invaded nematodes was observed using a microscope. Photographs at each inoculation period were taken under an Olympus SZX16 stereomicroscope.

### RNA extraction and Illumina sequencing

As documented above, an inoculation test was conducted, and root tips from IL10–1 and CC3 at 0, 1, 2 and 3 dpi (Rck, Rt1, Rt2, Rt3 and Sck, St1, St2, St3, respectively), were harvested. The root tips from 7 plants were mixed into a single biological sample, with 3 biological replicates from each sample. Then, the 24 total samples were immediately frozen in liquid nitrogen and stored at − 80 °C. Extraction of total RNAs were conducted using the TRIzol reagent (Invitrogen, Carlsbad, CA, USA). A total of 5 μg of RNA per sample was used as input material for the RNA sample preparations. Twenty-four cDNA libraries were generated using a NEBNext®UltraTM RNA Library Prep Kit for Illumina® (NEB, USA) following manufacturer protocol. The clustering of the index-coded samples was performed on a cBot Cluster Generation System using a TruSeq PE Cluster Kit v4-cBot-HS (Illumina) according to the manufacturer’s instructions. After cluster generation, the library preparations were sequenced on an Illumina Hiseq 2500 platform and paired-end reads were generated.

Clean data with high quality were obtained by removing reads containing adapters, reads containing ploy-N and low quality reads from raw data. After quality control with FastQC (http://www.bioinformatics.babraham.ac.uk/projects/fastqc/),reads were blasted to the ‘9930’ (v2) [58] cucumber genome using TopHat v2.0.12. The expression level of each gene based on RNA-seq was measured as numbers of reads per kilo base of exon region in a gene per million mapped reads (RPKM). The DESeq R package (1.18.0) was used to identify the differentially expressed unigenes(DEGs), with an adjusted false discovery rate (FDR) < 0.05 and |log2 FC (fold change)| ≥ 1, among the 24 libraries. The clean data from this work are deposited in the Sequence Read Archive (https://www.ncbi.nlm.nih.gov/sra/) under accession number SRP125669.

### Classification and functional annotation

To gain insight into the gene expression patterns, cluster analysis was performed by k-means based on inputs of log2 FC of the union of DEGs [59]. To analyze the function of each category, Gene Ontology (GO) enrichment was conducted to obtain GO annotation with the R package topGO [60]. GO terms with FDR-corrected *P*-values < 0.05 were considered significantly enriched by differentially expressed genes.

### Gene network construction

To improve the accuracy of network construction, the genes used for WGCNA (weighted gene co-expression network analysis) were filtered. The genes expressed in more than half of the samples extracted at 0, 1 and 3 dpi of both IL10–1 and CC3 were selected. Referring to the Steve H and Peter L method [61], based on FPKM and default settings (the power was 16 and mergeCutHeight was 0.25), modules were obtained using the automatic network construction function. The eigengene value was calculated for each module and used to test the association with each sample. Finally, Cytoscape_v.3.0.0 was used to transform the network into visual graphics.

### Quantitative analysis of IAA

Root tips of IL10–1 and CC3 were collected at 1, 2, and 3 dpi including inoculation and mock-inoculation using the method mentioned above. According to the experimental procedure with modification [62, 63], IAA was extracted from 200 mg of fresh weight root tissue, and then was purified and quantified using HPLC-MS/MS [64].

### Histological characterization

Galls of germinated seedlings infected by the RKN incubated as described above were acquired. All galls from IL10–1 and CC3 at 3 dpi were used for the histological study. According to the method of Sun, C. Q. [65], sample sections were sliced to 8–10 μm and stained with HE (hematoxylin eosin). Then, the sections were observed and photographed under an Olympus BX41 microscope.

### Validation of RNA-seq data by quantitative real time PCR (qPCR)

Based on different expression patterns, 12 DEGs were selected to verify the accuracy of the transcriptome results by qPCR. A housekeeping gene Csa6M484600 encoding ACTIN was used as the internal control [66]. Primers for qPCR were designed using Primer 6 software (Additional file 7: Table S4) and synthesized by Invitrogen. cDNA was synthesized from the total RNA used for RNA-seq using the PrimeScript RT reagent Kit (Takara), and then qPCR repeated three times was performed on Bio-Rad CFX96 using the 2× SYBR green PCR master mix (Applied Biosystems). The relative expression of each gene was calculated using the 2^− ΔΔCT^ method [67].

## Additional files

Additional file 1: **Table S1.** The statistics of RNA-seq sequencing data in this study. Rck and Rt represent the control and treatment, respectively, of IL10–1 and and Sck and St represent the control and treatment of CC3. 1, 2 and 3 represent three biological replicates. (DOCX 1112 kb)

Additional file 2: **Figure S1.** The heat map showing the correlations between samples. The colour represents the correlation coefficient between the two samples, and the deeper colour indicates a higher correlation coefficient. (DOCX 14 kb)

Additional file 3: **Figure S2.** Clustering of the gene expression profiles in IL10–1 and CC3 infected with *M. incognita* by *k*-means clustering method. R represents IL10–1; S represents CC3. (DOCX 16 kb)

Additional file 4: **Table S2.** GO enriched analysis of clusters of k-means. (DOCX 1396 kb)

Additional file 5: **Figure S3.** WGCNA of genes of IL10–1 at 3 dpi. (a) Hierarchical cluster tree. Each leaf in the tree is one gene; Each colour indicates a modules. DynamicColours: Module based on clustering results. MergedColours: The merged module according to the similarity of the modules (The correlation coefficient was greater than 0.75). (b) Module-sample relationships. Each row corresponds to a module. Each column corresponds to a specific sample. The colour of each square represents the correlation coefficient between the module and the sample, and the numbers in brackets indicate the *P*-value. (DOCX 776 kb)

Additional file 6: **Table S3.** The correlation network of the MEhoneydew module. Identification of the 259 genes with a raised weighted cutoff value > 0.16. (XLSX 38 kb)

Additional file 7: **Table S4.** List of primers used in quantitative RT-PCR for validation of RNA-Seq analysis. All primers were designed by Primer 6. (XLSX 191 kb)

## Abbreviations

DEG: Differentially expressed gene
dpi: Day post inoculation
FDR: False discovery rate
GC: Giant cell
GO: Gene ontology
HR: Hypersensitive response
IAA: Indoleacetic acid
LTP: Lipid transfer protein
NFS: Nematode feeding sites
PR: Pathogenesis-related
RKN: Root-knot nematode
ROS: Reactive oxygen species
SAR: Systemic acquired resistance
