# Supplementary material for: Comparative transcriptomics reveals suppressed expression of genes related to auxin and the cell cycle contributes to the resistance of cucumber against Meloidogyne incognita
Source: BMC Genomics. 2018 Aug 3;19:583. doi: 10.1186/s12864-018-4979-0 (PMC6090858; doi:10.1186/s12864-018-4979-0)
Supplement: Supplementary file 1 — Table S1. The statistics of RNA-seq sequencing data in this study. Rck and Rt represent the control and treatment, respectively, of IL10–1 and and Sck and St represent the control and treatment of CC3. 1, 2 and 3 represent three biological replicates. (DOCX 1112 kb) [file 12864_2018_4979_MOESM1_ESM.docx]

| **Sample** | **Replicates** | **Clean Reads** | **Total Mapped Reads** | **Unique Match** | **% Unique alignment** |
| --- | --- | --- | --- | --- | --- |
| IL10-1-0d-1（Rck） | 1 | 43,825,770 | 26,780,254 | 26,392,200 | 98.55% |
| IL10-1-0d-2（Rck） | 2 | 48,091,758 | 28,134,662 | 27,746,450 | 98.62% |
| IL10-1-0d-3（Rck） | 3 | 47,166,950 | 27,210,891 | 26,842,506 | 98.65% |
| IL10-1-1d-1（Rt1） | 1 | 46,699,014 | 27,408,165 | 27,033,113 | 98.63% |
| IL10-1-1d-3（Rt1） | 2 | 46,558,398 | 27,003,140 | 26,632,444 | 98.63% |
| IL10-1-1d-4（Rt1） | 3 | 46,185,830 | 26,769,568 | 26,406,208 | 98.64% |
| IL10-1-2d-1（Rt2） | 1 | 45,618,402 | 26,277,885 | 25,939,823 | 98.71% |
| IL10-1-2d-3（Rt2） | 2 | 46,960,688 | 26,905,622 | 26,549,265 | 98.68% |
| IL10-1-2d-4（Rt2） | 3 | 45,987,526 | 26,154,167 | 25,799,724 | 98.64% |
| IL10-1-3d-1（Rt3） | 1 | 46,941,966 | 27,028,406 | 26,670,822 | 98.68% |
| IL10-1-3d-3（Rt3） | 2 | 45,908,182 | 25,876,384 | 25,539,577 | 98.70% |
| IL10-1-3d-4（Rt3） | 3 | 46,700,660 | 26,698,916 | 26,346,317 | 98.68% |
| CC3-0d-1（Sck） | 1 | 45,847,062 | 26,621,476 | 26,191,313 | 98.38% |
| CC3-0d-2（Sck） | 2 | 46,686,806 | 27,761,056 | 27,297,001 | 98.33% |
| CC3-0d-3（Sck） | 3 | 46,327,210 | 27,065,418 | 26,658,355 | 98.50% |
| CC3-1d-1（St1） | 1 | 46,943,678 | 27,061,171 | 26,710,400 | 98.70% |
| CC3-1d-2（St1） | 2 | 46,527,256 | 27,060,637 | 26,704,175 | 98.68% |
| CC3-1d-3（St1） | 3 | 46,697,328 | 27,125,689 | 26,744,568 | 98.59% |
| CC3-2d-2（St2） | 1 | 47,161,856 | 26,904,833 | 26,490,327 | 98.46% |
| CC3-2d-3（St2） | 2 | 48,041,460 | 27,493,834 | 27,100,124 | 98.57% |
| CC3-2d-4（St2） | 3 | 43,575,552 | 26,098,271 | 25,733,554 | 98.60% |
| CC3-3d-1（St3） | 1 | 45,231,890 | 27,128,296 | 26,725,727 | 98.52% |
| CC3-3d-3（St3） | 2 | 41,499,404 | 24,708,241 | 24,334,384 | 98.49% |
| CC3-3d-4（St3） | 3 | 47,197,008 | 28,276,181 | 27,880,628 | 98.60% |

**Table S1.** The statistics of RNA-seq sequencing data in this study. Rck, Rt and Sck, St represent the control and the treatment of IL10-1 and CC3. 1, 2, 3 represent three biological replicates.
