# Supplementary material for: Comparative transcriptomics reveals suppressed expression of genes related to auxin and the cell cycle contributes to the resistance of cucumber against Meloidogyne incognita
Source: BMC Genomics. 2018 Aug 3;19:583. doi: 10.1186/s12864-018-4979-0 (PMC6090858; doi:10.1186/s12864-018-4979-0)
Supplement: Supplementary file 2 — Figure S1. The heat map showing the correlations between samples. The colour represents the correlation coefficient between the two samples, and the deeper colour indicates a higher correlation coefficient. (DOCX 14 kb) [file 12864_2018_4979_MOESM2_ESM.docx]

**
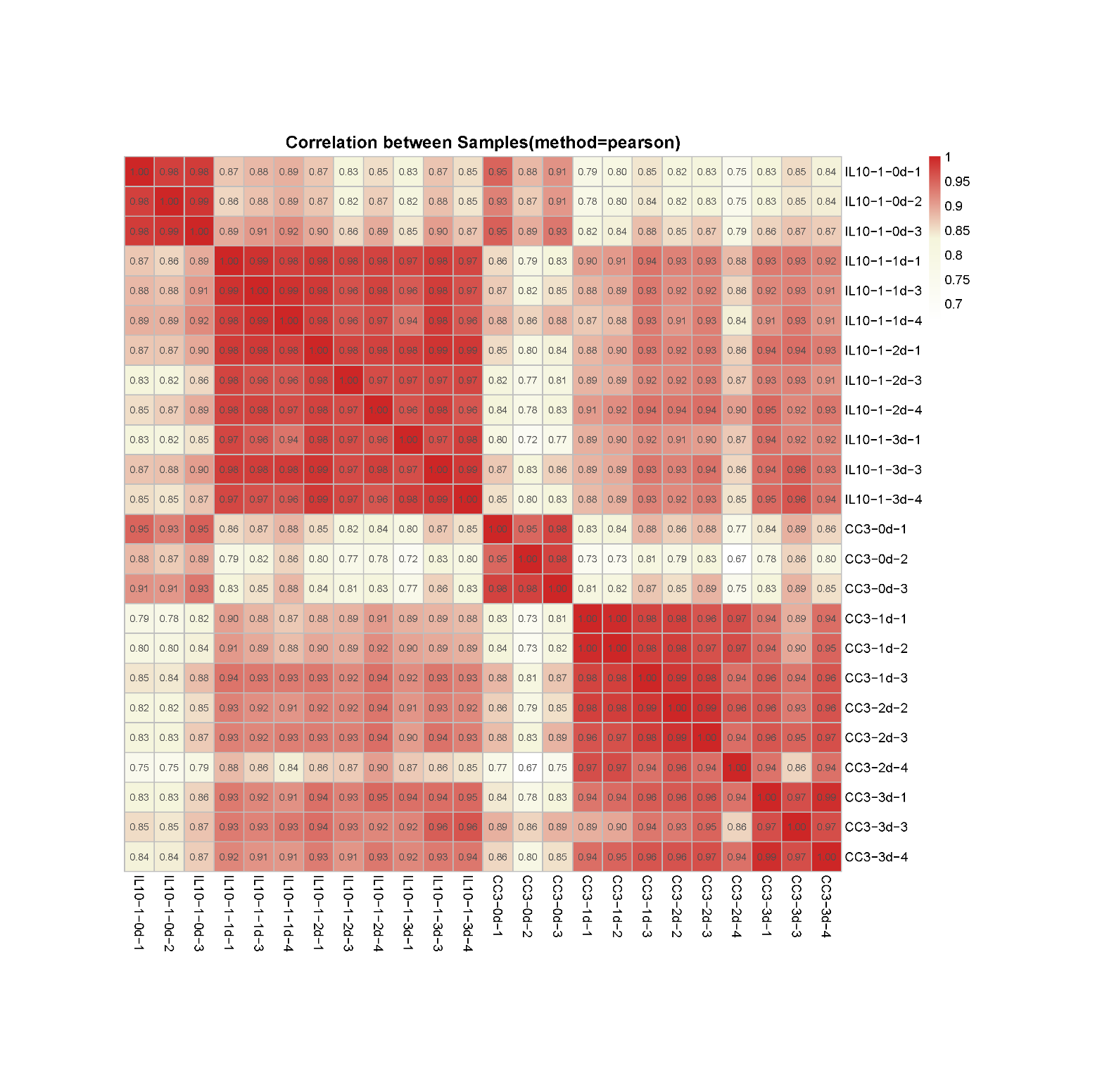
**

**Figure S1.** The heat map showing the correlation between samples. The color represents the correlation coefficient between the two samples, The deeper the color, the greater the correlation coefficient.
