# Supplementary material for: Comparative transcriptomics reveals suppressed expression of genes related to auxin and the cell cycle contributes to the resistance of cucumber against Meloidogyne incognita
Source: BMC Genomics. 2018 Aug 3;19:583. doi: 10.1186/s12864-018-4979-0 (PMC6090858; doi:10.1186/s12864-018-4979-0)
Supplement: Supplementary file 3 — Figure S2. Clustering of the gene expression profiles in IL10–1 and CC3 infected with M. incognita by k-means clustering method. R represents IL10–1; S represents CC3. (DOCX 16 kb) [file 12864_2018_4979_MOESM3_ESM.docx]

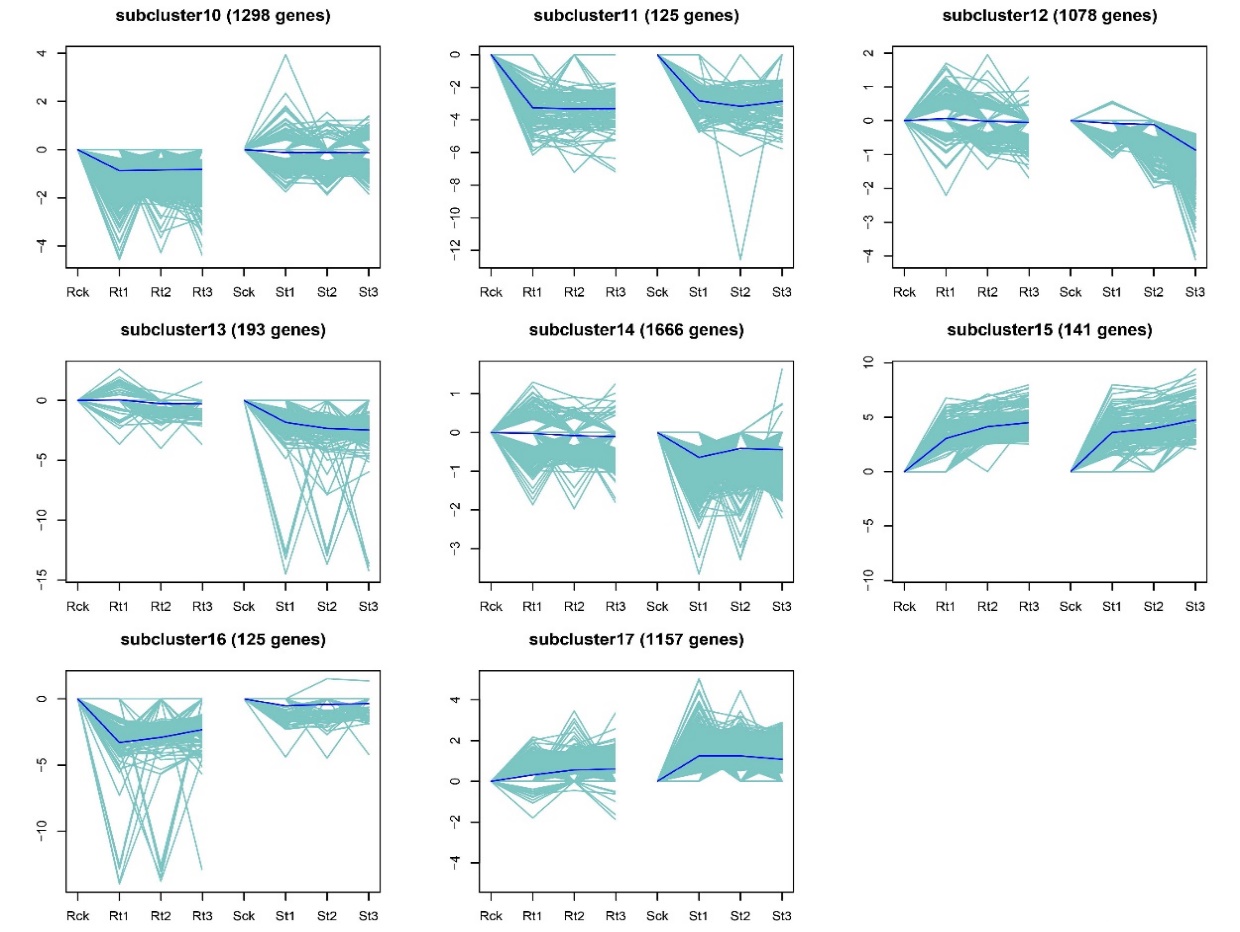

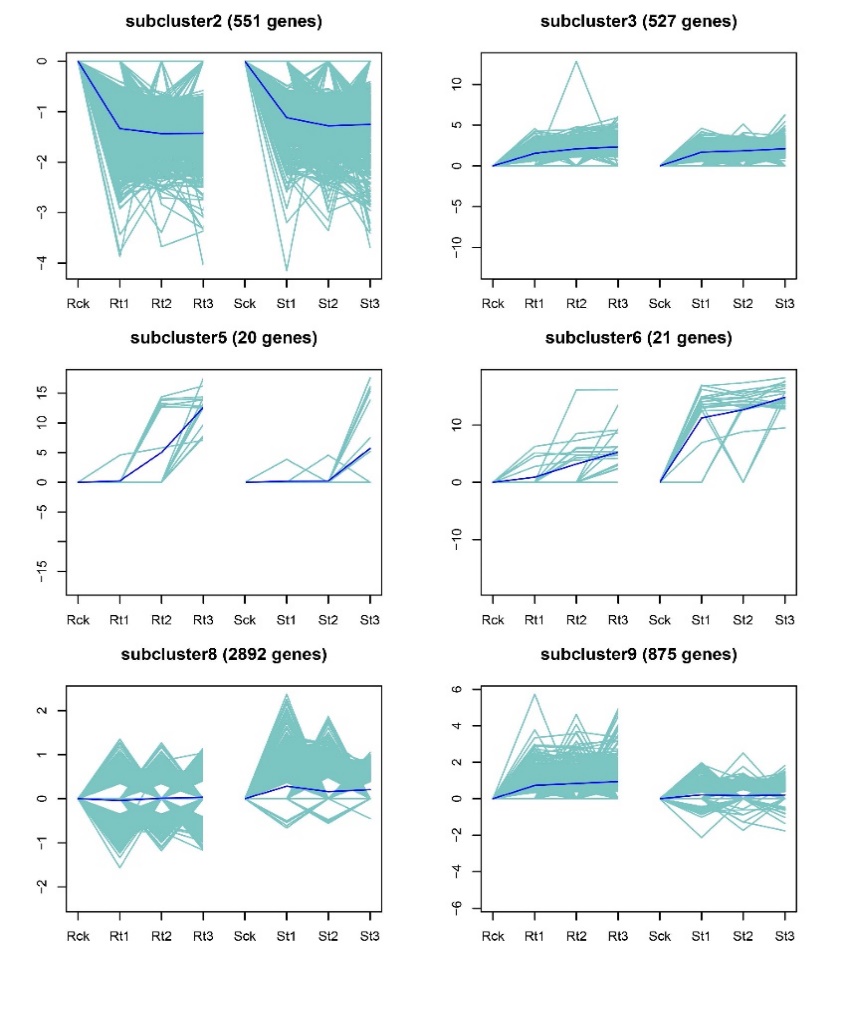

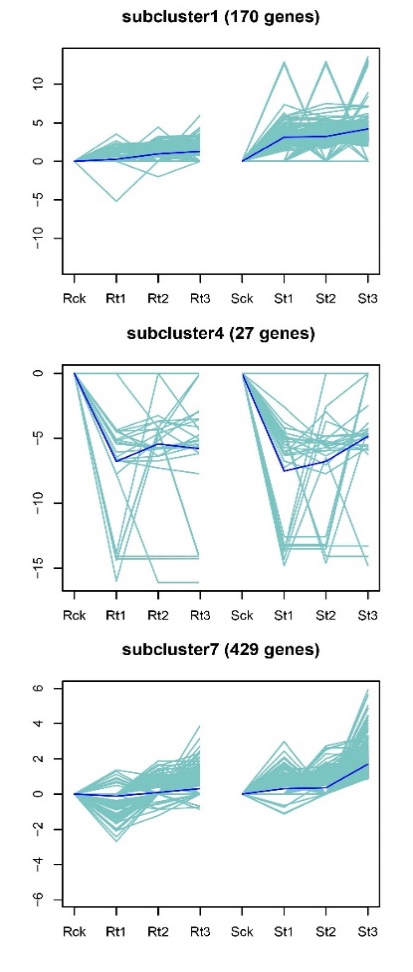


**Figure S2.** Clustering of the gene expression profiles in IL10-1 and CC3 infected with the *M. incognita* by *k*-means clustering method. R represent IL10-1, S represent CC3.
