# Supplementary material for: Comparative transcriptomics reveals suppressed expression of genes related to auxin and the cell cycle contributes to the resistance of cucumber against Meloidogyne incognita
Source: BMC Genomics. 2018 Aug 3;19:583. doi: 10.1186/s12864-018-4979-0 (PMC6090858; doi:10.1186/s12864-018-4979-0)
Supplement: Supplementary file 5 — Figure S3. WGCNA of genes of IL10–1 at 3 dpi. (a) Hierarchical cluster tree. Each leaf in the tree is one gene; Each colour indicates a modules. DynamicColours: Module based on clustering results. MergedColours: The merged module according to the similarity of the modules (The correlation coefficient was greater than 0.75). (b) Module-sample relationships. Each row corresponds to a module. Each column corresponds to a specific sample. The colour of each square represents the correlation coefficient between the module and the sample, and the numbers in brackets indicate the P-value. (DOCX 776 kb) [file 12864_2018_4979_MOESM5_ESM.docx]

**
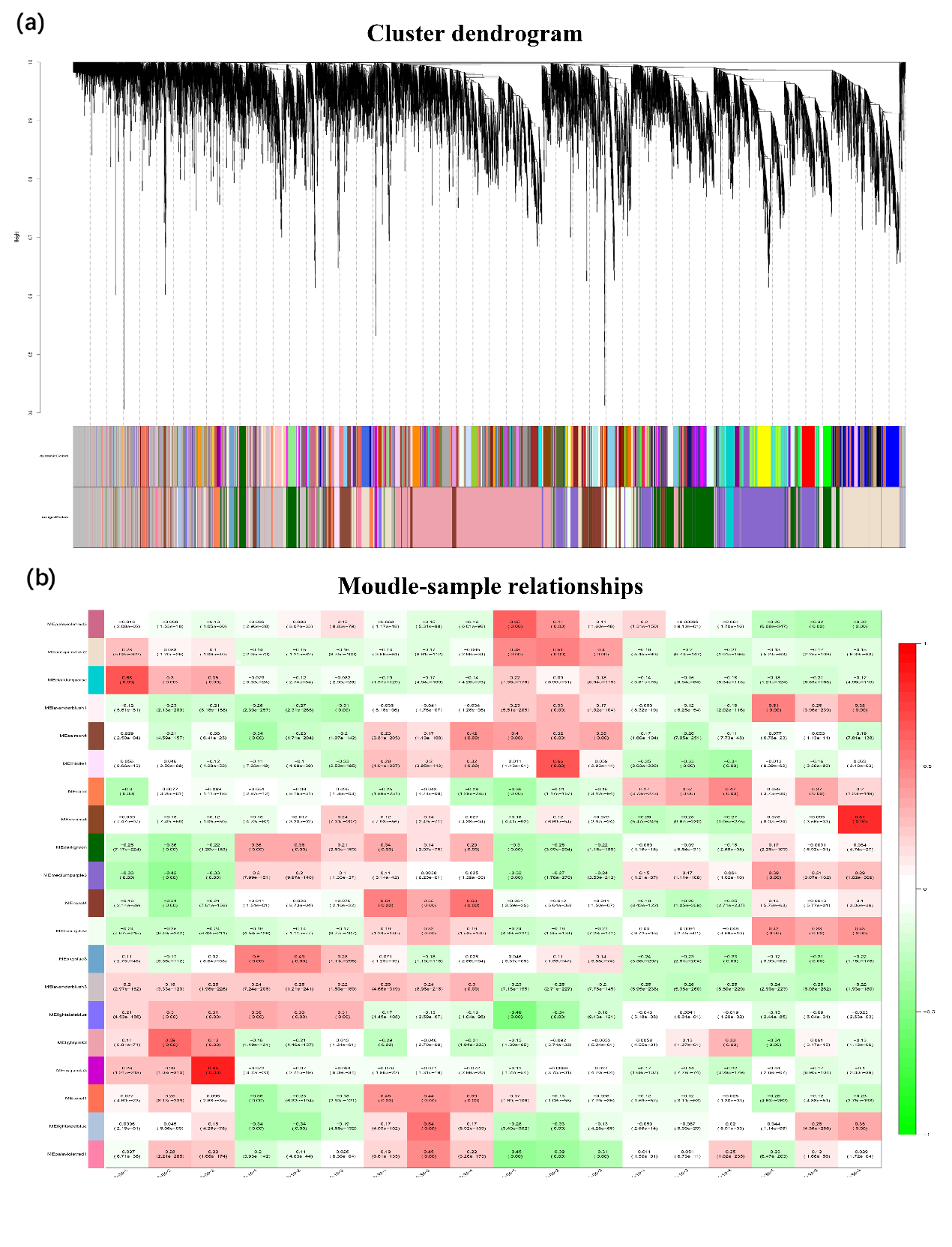
**

**Figure S3.** WGCNA of genes of IL10-1 at 3dpi. **(a)** Hierarchical cluster tree. Each leaf in the tree is one gene, Each color means a modules. DynamicColors: Module based on clustering results. MergedColors: The merged module according to the similarity of the module (The correlation coefficient is greater than 0.75). **(b)** Module-sample relationships. Each row corresponds to a module. Each column corresponds to a specific sample. The color of each square represents the correlation coefficient between the module and the sample, the numbers in brackets means *p*-value.
