# Supplementary material for: Comparative transcriptomics reveals suppressed expression of genes related to auxin and the cell cycle contributes to the resistance of cucumber against Meloidogyne incognita
Source: BMC Genomics. 2018 Aug 3;19:583. doi: 10.1186/s12864-018-4979-0 (PMC6090858; doi:10.1186/s12864-018-4979-0)
Supplement: Supplementary file 7 — Table S4. List of primers used in quantitative RT-PCR for validation of RNA-Seq analysis. All primers were designed by Primer 6. (XLSX 191 kb) [file 12864_2018_4979_MOESM7_ESM.xlsx]

| **Gene ID** | **Sense Primer** | **Anti-sense Primer** |
| --- | --- | --- |
| Actin ( Csa6M484600) | GGAGAAGATCTGGCATCACA | CTCCAATCCAGACACTGTACT |
| Csa7G070235 | CCTCCTGGCTACTACCATCTC | GCATAGGCGGCTAGAGTGT |
| Csa7G072220 | CCTTGCCATGTCTTCCTTGTC | GAGTGTGGTGGAGATGGTGAT |
| Csa7G072840 | GTTGTCGTCGTTGTCGCTAC | CACCGCCTGAGAAGAATGC |
| Csa3G018320 | AGGAGGAGGTGAAGCAAGTG | ACGGTGATAACGGCGGAATA |
| Csa1G001290 | CTGTCATGTGGCTCTGCTATG | CGGCTTGGCGTTTCTGTAG |
| Csa5G601470 | CTCTTGTGGTGACGACTGTATT | AGACTTCCAATGCTGATTCCAT |
| Csa5G167120 | CGTAGCGATTCCACCTAAGAG | GGCGAACTCCTCTGTAATGC |
| Csa3G895090 | CCTGTCTTAGTGCTTCCTTACC | AATCCAAGTTCTGAGGCATCAA |
| Csa6G216960 | GCCGTTGTCTCCACCACTT | GGCGATAGCAGAATACGAGAAG |
| Csa1G383520 | ACACTCCTACGGTTCCTCCT | GAATGGTGGTGATGATGTTCCT |
| Csa3G171210 | CCAAGATACCAGCAGCAGATG | AGAGATCCTCCAACTGAAGTGA |
| Csa2G049330 | GAGTACCCTTCCCACAATATCA | GGCTCCAATCTGTCTTCTCAA |

**Table S4** List of primers used in quantitative RT-PCR for validation of RNA-Seq analysis. All primers are designed by Primer 6.
